# Guideline for Limb‐Salvage Treatment of Osteosarcoma

**DOI:** 10.1111/os.12702

**Published:** 2020-07-06

**Authors:** Ming Xu, Zhen Wang, Xiu‐chun Yu, Jian‐hua Lin, Yong‐cheng Hu

**Affiliations:** ^1^ Department of Orthopedics The 960th Hospital of PLA Jinan China; ^2^ Department of Orthopedics Xi‐jing Hospital, Air Force Military Medical University Xi'an China; ^3^ Department of Orthopedics The 960th Hospital of PLA Jinan China; ^4^ Department of Orthopedics The First Affiliated Hospital of Fujian Medical University Fuzhou China; ^5^ Department of Bone Oncology Tianjin hospital Tianjin China

**Keywords:** Limb‐salvage therapy, Neoadjuvant chemotherapy, Osteosarcoma

## Abstract

Osteosarcoma is the most common primary malignant bone tumor, occurring mainly in children and adolescents, and the limbs are the main affected sites. At present, limb‐salvage treatment is considered as an effective basic standard treatment for osteosarcoma of the limb. China has a vast territory, but the development of technology is not balanced,which requires sufficient theoretical coverage, strong technical guidance and the application of limb‐salvage treatment guidelines to the treatment of osteosarcoma. Therefore, to standardize and promote the development of limb‐salvage surgery technology and improve the success rate of limb‐salvage treatment, this guide systematically introduces limb‐salvage techniques for the treatment of patients with limb osteosarcoma through definition of limb‐salvage treatment, surgical methods, efficacy evaluation, postoperative treatment and prevention of complications, rehabilitation guidance, and follow‐up advice.

## Background

Osteosarcoma is the most common malignant bone tumor, which occurs with a high degree of malignancy and rate of disability in adolescents. The 5‐year survival rate of the natural course of the disease is only 10%–20%. Classic osteosarcoma, or conventional osteosarcoma, is the most common subtype of osteosarcoma, accounting for 80% of all osteosarcomas. It usually occurs at the distal end of the femur and proximal end of the tibia^1^, ^2^.

In the past 30 years, significant progress has been made in China in the diagnosis and treatment techniques for osteosarcoma. Surgery is recognized as an effective basic treatment for primary and recurrent metastatic osteosarcoma. The 5‐year survival rate of conventional osteosarcoma has been significantly improved, with the tumor‐free survival rate reaching 60%–70% and the overall survival rate reaching 60%–80%^3^, ^4^, ^5^. Limb‐salvage treatment has become one of the standard treatment methods for patients with limb osteosarcoma, with 90% of patients undergoing limb‐salvage surgery and a success rate of 60%–80%^3^, ^4^, ^5^, ^6^.

All patients with suspected osteosarcoma should be referred to the osteosarcoma diagnosis and treatment center or institutions with a specialized osteosarcoma diagnosis and treatment system before biopsy, and the hospitals that implement the limb‐salvage treatment should have a team of experts in imaging, pathology, intervention, and other disciplines for the diagnosis of bone tumors, as well as a specialized department for bone tumor and an oncology department with experience in chemotherapy for osteosarcoma. The attending doctor is responsible for implementing the overall treatment plan of the patient and multidisciplinary team consultation^7^, ^8^.

China is a vast territory, but access to technological advances is not equitable across all regions of the country, so it is necessary to provide sufficient theoretical coverage and strong technical guidance as well as apply limb‐salvage treatment guidelines to the treatment of osteosarcoma. Accordingly, in April 2018, members of the bone oncology group of the Chinese Orthopaedic Association jointly discussed the relevant information and developed this guide in accordance with the principles of science, practicability, and progressiveness^9^. The purpose of these guidelines is to standardize and promote the methods of limb‐salvage surgery for osteosarcoma and improve the success rate of limb‐salvage treatment for osteosarcoma. This guide is applicable to the multidisciplinary healthcare team, including orthopaedic surgeons, bone oncologists, oncology physicians, and doctors, involved in the diagnosis and treatment of the conventional primary osteosarcoma of the long bone of the extremities.

## Definition

### *Limb‐Salvage Therapy*

Limb‐salvage therapy refers to a series of treatments, such as neo adjuvant chemotherapy, limb‐salvage surgery, and adjuvant chemotherapy, which are completed through the joint efforts of a multidisciplinary team of doctors. The purposes of limb‐salvage treatment are to reduce local recurrence as much as possible to retain good limb function and improve the survival rate of patients^10^, ^11^, ^12^.

### *Limb‐Salvage Surgery*

Limb‐salvage surgery refers to the surgical procedure to restore bone and joint function after extensive resection of malignant bone tumors of the limbs.

### *Neoadjuvant Chemotherapy*

Neoadjuvant chemotherapy refers to the preoperative chemotherapy after the definite diagnosis of malignant bone tumors^11^, ^12^, ^13^, ^14^, ^15^, ^16^.

### *Tumor Margin*

Tumor margin refers to the continuous integrity of the normal tissue adjacent to the tumor and the reaction area. The design of the surgical margin depends on the tumor margin, which has a barrier effect to prevent local invasion of the tumor and is a key factor in determining the prognosis^17^, ^18^.

### *Surgical Margin of the Tumor*

The surgical margin of the tumor refers to the actual extent of the tumor specimen to be removed. Extensive resection refers to the removal of the area of normal tissue surrounding the tumor response layer. After extensive resection, the local control rate of the tumor can reach more than 90%. The aim of surgical excision for limb‐salvage is to minimize the loss of normal tissue while obtaining a safe and tumor‐free surgical margin, so as to retain the best function of the limb^18^, ^19^, ^20^, ^21^.

### *Safety Margin*

Safety margin refers to the normal bone tissue in the longitudinal and/or transverse membranous and medullary cavity 3–5 cm away from the margin of the tumor, as well as the fascia, tendon, joint capsule, articular cartilage, blood vessel, nerve sheath, and outer membrane that are not invaded by the tumor, and where no tumor tissue was detected under the microscope^22^.

## Diagnosis of Osteosarcoma

The diagnosis of osteosarcoma of limb bones mainly relies on a combination of clinical, imaging, and pathological examination. The relatively constant cooperation of the multidisciplinary team participating in the diagnostic process can make the diagnosis and differential diagnosis more accurate. The composition of multidisciplinary teams and the division of tasks are shown in Table 1. Definitive diagnosis and staging of the tumor are required prior to chemotherapy.

**TABLE 1:** Tasks and division of labor of multidisciplinary medical team for limb‐salvage treatment of osteosarcoma

Medical team	Diagnosis and staging	Preoperative treatment plan	New adjuvant therapy	Operation	Sample evaluation	Postoperative treatment	Adjuvant therapy	Monitoring and follow‐up
Oncologist	√	√	√	√	√	√	‐	√
Image doctor	√	√	‐	‐	√	‐	‐	√
Pathologist	√	√	‐	‐	√	‐	‐	‐
Medical Oncologist	‐	√	√	‐	‐	‐	√	√
Interventional or radiotherapy doctor	√	√	√	√	‐	√	√	√
Professionals in specific fields	‐	√	‐	√	‐	√	√	√

Note: Diagnosis stage: imaging evaluation, biopsy; Preoperative diagnosis and treatment plan: edge design, preoperative embolization, image fusion, three‐dimensional (3D) printing, prosthesis; Neoadjuvant chemotherapy treatment: preoperative chemotherapy, radiotherapy; Operation: primary tumor resection, bone and joint function reconstruction, vascular embolism, metastasis resection, skin coverage, vascular reconstruction; Sample evaluation: surgical edge tumor necrosis rate; Postoperative treatment: thrombus prevention and treatment, perioperative rehabilitation; Adjuvant treatment: chemotherapy, radiotherapy, psychotherapy; Monitoring and follow‐up: tumor control, postoperative rehabilitation, routine monitoring, data processing; Professionals in specific fields: image fusion, 3D printing, prosthesis design, psychotherapy, postoperative rehabilitation, related surgery.

### *Imaging Study*

1. X‐ray: AP and lateral X‐ray of the primary lesion.
2. Computed tomography (CT, enhancement): lesions and chest; chest examination require thin layer + coronal position.
3. Magnetic resonance imaging (MRI) examination: weighted T1 and T2 and enhanced MRI.
4. Bone scan: whole‐body + radioactive concentration region in TomoScan.
5. It should be noted that positron emission tomography (PET), CT, and MRI examinations are optional^10^.

### *Biopsy*

Biopsy is necessary for definitive diagnosis before treatment, and coarse needle biopsy is recommended^3^, ^4^, ^7^, ^8^, ^11^. Subsequent limb salvage and reconstruction should be considered during biopsy. The biopsy needle tract should be as close as possible to the planned surgical incision and should be excised entirely with the tumor at the time of final surgery, without crossing the tumor‐free anatomic compartment, joint, and neurovascular tract. The epiphysis must not be passed through the biopsy needle in adolescent patients. In addition, if there are multiple lesions, easily accessible sites for biopsy should be chosen.

## Limb Salvage for Osteosarcoma

**Fig 1:**
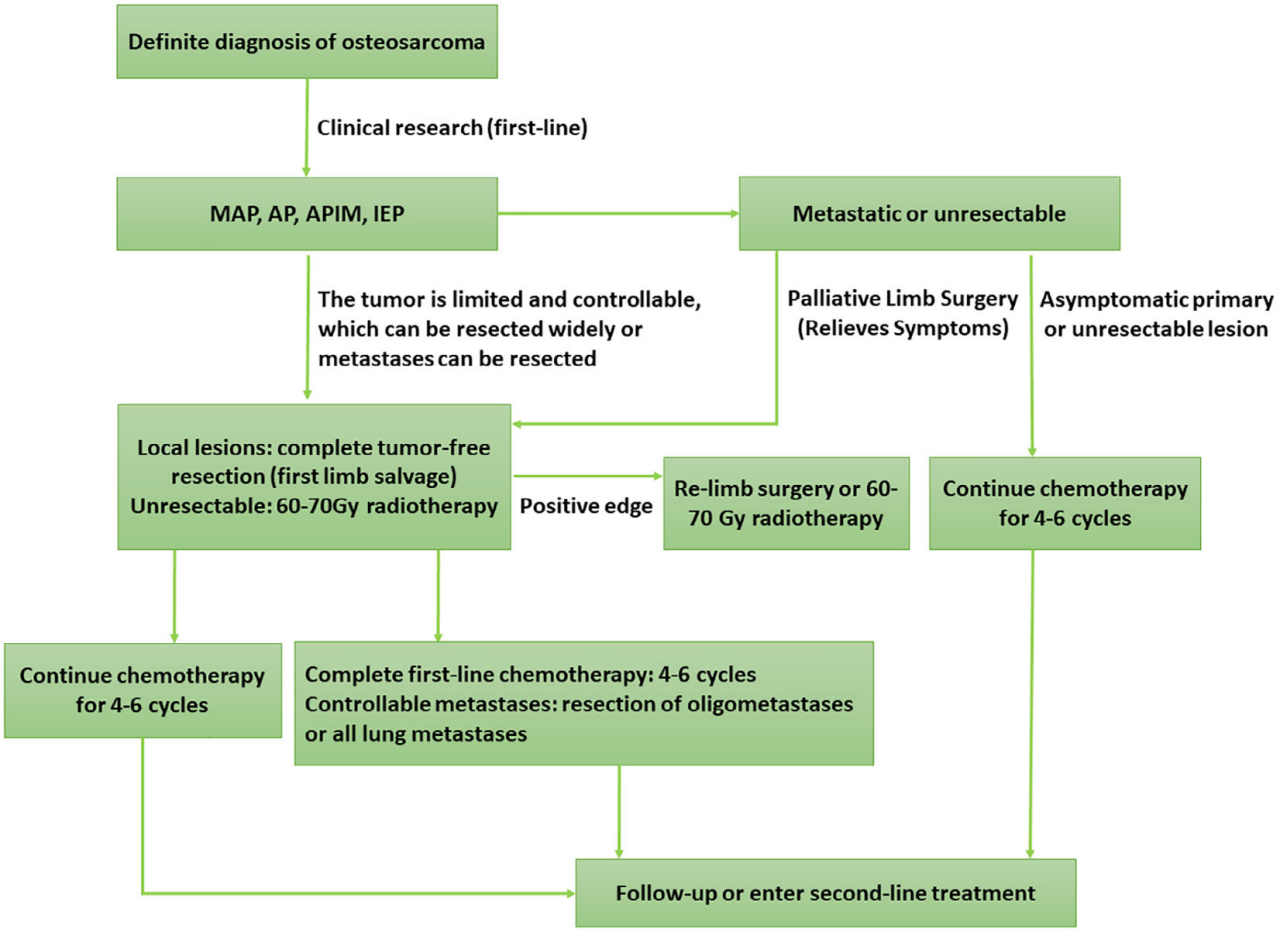
Flow chart of the limb‐salvage treatment for conventional osteosarcoma of the limb. Frontline: Including initial, neo‐adjuvant, and adjuvant chemotherapy, which are generally proven to have stable and reliable efficacy by evidence‐based medicine, and are recognized by most experts. First‐line treatment options are not static. Second‐line: The chemotherapy regimen used for first‐line treatment failure, including relapse, refractory or metastatic osteosarcoma, has limited efficacy. MAP, AP, APIM, and IEP represent different neoadjuvant chemotherapy regimens, respectively.

Typical limb‐salvage treatment methods for patients with osteosarcoma include neoadjuvant chemotherapy, limb‐salvage surgery, and adjuvant chemotherapy. The specific treatment process is shown in Fig. 1.

### *Neoadjuvant Chemotherapy*

#### *The Purpose of Neoadjuvant Chemotherapy*

Besides controlling the primary lesion and killing distant micro metastases as soon as possible, the purpose of neoadjuvant chemotherapy is to reduce the tumor and surrounding inflammatory edema reaction area, in order to facilitate subsequent limb‐salvage surgery. The sensitivity of the tumor to chemotherapy was found to lay the foundation for further development of individualized postoperative chemotherapy regimens^3^, ^4^, ^11^, ^12^, ^13^, ^14^, ^15^, ^16^, ^23^.

Neoadjuvant chemotherapy also has risks. For example, some patients have more lesions and/or reduced physical health after receiving neoadjuvant chemotherapy, making radical tumor resection impossible.

#### *Principles of Drug Use in Non‐Adjuvant Chemotherapy*

In sequential or combined use, each patient should choose at least two or more drugs to be administered intravenously or intravenously according to the instructions. The initial dose is calculated according to the dose of the standard regimen, and the total dose intensity is maintained as long as possible. Under the premise of closely observing the effect of chemotherapy, it is recommended to use each drug for at least two cycles. Interval medication is required according to the selected standard regimen.

#### *Drugs Used in Neoadjuvant Chemotherapy*

Tetracycline [ADM, PLD, THP, EPI], Platinum [DDP, LBP], HDMTX‐*CF*, IFO.

#### *Chemotherapy Regimens for Non‐Adjacent Chemotherapy*

Common non‐adjacent chemotherapy regimens:

AP regimen (Adriamycin [ADM] 75 mg/m^2^ d1 + Cisplatin [DDP] 75 mg/m^2^,Q3W).

MAP regimen (high dose methotrexate [HDMTX] 8–10 g/m^2^ d1 + ADM 60 mg/m^2^ d1 + DDP 75 mg/m^2^,Q3W).

DIA regimen (DDP 100 mg/m^2^ d1+ Ifosfamide [IFO] 2 g/m^2^ d7‐d11+ ADM 30 mg/m^2^ d7‐d11, Q3W)^24^.

APIM regimen (ADM 60 mg/m^2^ d1 + DDP 75 mg/m^2^ + IFO 1.8 g/m^2^ d1‐d4 + HDMTX 8–10 g/m^2^ d1,Q3W).

IEP regimen (Epirubicin [EPI] 90mg/m^2^ d1 + DDP 100 mg/m^2^ d1 + IFO 2 g/m^2^ d2‐d4,Q3W)^25^ ^.^

##### Evaluation After Chemotherapy

After completion of non‐adjacent chemotherapy, the tumor should be evaluated again in detail in combination with clinical symptoms, signs, and imaging examination. More than two doses or at least one cycle are recommended for evaluation.

Good clinical outcome in response to chemotherapy: clinical symptoms were reduced, the imaging boundaries of the tumor were clear, the tumor tissues showed ossification and tumor shrinkage^2^, ^7^, ^8^. Limb salvage should be the first choice for patients with high‐grade osteosarcoma who respond well to chemotherapy if they can reach a broad surgical boundary, as limb‐salvage surgery can prevent the psychological impact caused by disability^6^, ^26^. Amputation should be performed only when limb‐salvage surgery cannot reach adequate surgical boundaries. For patients with metastases, amputation is generally not recommended since radical treatment has not been achieved.

### *Limb‐Salvage Surgery*

#### *Preoperative Preparation and Timing of Surgery*

It is recommended to use the Enneking staging system and AJCC / UICC staging system before surgery. The purpose is to point out the relative risk of different surgical procedures in case of a certain lesion.

Combining tumor staging, surgical skills and experience of doctors, with the effect of neoadjuvant chemotherapy, as well as with the opinions of the members of the multidisciplinary team, the preoperative preparation was completed (Table 1).

It is suggested that the operation should be performed within 3 weeks after the end of chemotherapy^27^. During chemotherapy, when the tumor grows to over 30% of the maximum diameter and breaks through the pseudocapsule, the administration of the drug should be stopped and the surgery should be performed. In order to locally control the tumor and relieve symptoms, radical resection is recommended.

#### *Indications for Limb‐Salvage Surgery*

Enneking stage IIA, stage II B sensitive to chemotherapy, stage III sensitive to chemotherapy, and metastases controllable.

Good chemotherapy response with pathological fracture of the limbo osteosarcoma.

The surgical margin of extensive excision can be or is expected to be achieved.

The main vascular nerve is not involved.

Overall condition is good, with a physical condition score (Karnofsky score) >60.

There is a strong desire to retain limbs and limb functions.

Good soft tissue coverage^10^, ^28^, ^29^, ^30^.

#### *Principles of Tumor Resection in Limb‐Salvage Surgery*

En bloc resection and complete resection of bone and soft tissue containing the tumor, that is, complete sleeves of normal muscle and soft tissue, and interpretative visual thickness not less than 1 cm (based on MRI) are, albeit, still controversial. The safe margin of bone resection is 3 cm away from the tumor margin as shown by MRI^17^, ^31^, ^32^, ^33^. Together with the biopsy incision and the surrounding tissue of the biopsy tract, the tumor is often removed in its entirety. The procedure of resection strictly follows principles of tumor‐free techniques^26^. The main neurovascular bundles must be separated and protected, and the surgical margins adjacent to the important vascular nerve bundles must be tumor‐free (R0, microscopically negative).

Avoiding local recurrence is the standard for successful limb‐salvage surgery^19^, ^20^, ^21^, ^34^.

Bone and joint reconstruction surgery is a surgical treatment based on the safe edge, allowing the combined application of a variety of reconstruction techniques. When the tumor enters epiphysis or an adjacent joint, the joint and joint capsule need to be excised. Local muscle flap reconstruction and adequate coverage of the wound with normal soft tissue should be considered.

The lower limbs of children can be extended 1–1.5 cm at a time, as far as possible to take into account the growth and development potential of children^35^, ^36^, ^37^.

It is recommended to apply the pre‐surgical design based on digital technology, which is conducive to the accurate resection of the tumor^32^, ^38^, ^39^, ^40^, ^41^, ^42^, ^43^.

#### *Methods of Limb‐Salvage Surgery*

Limb‐salvage surgery allows the use of a variety of reconstruction techniques in combination or on their own, including tumor endoprostheses, autologous or extended allograft bone reconstruction, and soft tissue reconstruction at the site of tumor resection.

(i) Tumor endoprosthesis

Tumor endoprosthesis is the most commonly used technique for limb‐salvage reconstruction^28^, ^44^, ^45^. After excision of the tumors around the knee joint in mature adolescents or adults, it is recommended to choose the rotating hinged custom prosthesis or the assembled prosthesis. In addition, bone cement or cementless fixation should be selected according to the basic condition of the patient's bones^45^. In general, bipolar hemiarthroplasty replacement is selected for the proximal femoral prosthesis. For tumors of the proximal humerus, Malawer type I resection is a commonly used surgical resection method. It is recommended to use a half shoulder prosthesis for reconstruction. For other rare sites, an individual design should be chosen. Furthermore, clinical studies have been reported on the use of prosthetics with repair segments or block metal three‐dimensional (3D)‐printed prostheses in limb‐salvage therapy, which is recommended to be used in clinical research at qualified hospitals^38, 39^. The literature reports that the 5‐year survival rate of patients with tumor prosthesis of upper limbs is 85%–89.7% and that of lower limbs is 69%–78%, with a revision rate of 34%–40%^29^, ^46^, ^47^.

(ii) Autograft bone or large segment allograft bone reconstruction

Reconstruction of bone defects after tumor resection using autograft and/or allograft bone, with long‐term reliability bone reconstruction relying on bone‐to‐bone healing, including joint preserving reconstruction and joint fusion.

1. Long segment allograft: the limb‐salvage rate can reach 90%. The reconstruction success rate is related to the reconstruction site, and segment reconstruction success rate can reach 82%–84%^41^.However, this method has the risk of rejection reaction, allograft bone fracture, infection, and non‐union. Fifty‐four per cent of patients will require surgery again due to complications. Current clinical studies have demonstrated that osteoarticular allograft of the distal femur or the proximal tibia leads to more complications (60%), while lower‐limb weight‐bearing bone segment grafting is preferred^48^.
2. Inactivated reconstruction: tumor segment is inactivated *in vitro* or *in vivo* and then replanted *in situ*. It is generally considered to be an autologous bone graft. There are many inactivation methods, and there is no evidence of high‐level efficacy for most of them. Relatively, there is more clinical evidence for radio‐inactivation and freeze inactivation. The rate of bone end healing after treatment is 88%, which is higher than that of allograft, and has advantages in anatomical matching and soft tissue attachment^49^.However, the complications of this method are a concern, with a single center reporting an infection rate of 13%, a local recurrence rate of 9.6%, and a fracture incidence of 20% with radio‐inactivated bone^50^, ^51^.
3. Allograft bone or inactivated bone combined with artificial joint: this approach can reduce the complications of articular cartilage degeneration caused by osteoarticular allograft, and is beneficial to soft tissue adhesion. It has advantages in limb‐salvage reconstruction of the proximal humerus, femur, and tibia. The complication rate is 23%^52^, ^53^, ^54^, ^55^.
4. Fibula transplantation: it is recommended that free fibula with blood vessels be combined with other repair materials to reconstruct long segmental bone defects, especially for lower limb long bone resection with a length of more than 15cm, in patients who are over the age of 18. Using allograft bone, or other biomaterial segment grafts, combined with free‐fibula grafts with blood vessels can significantly reduce the incidence of complications^56^.The success rate of the allograft bone combined with free‐fibula grafts with blood vessels was 93.5%. However, this method has the risk of surgical complications in the donor area, non‐union of allograft bone, fracture of allograft bone, etc.^56^
5. Bone transport: For children who need to preserve epiphyses or joints can benefit, but indications are limited. Long segment bone transport may lead to complications, such as pin tract infection, limited movement of adjacent joints, and non‐union of bones^48^, ^54^.

(iii) Soft tissue reconstruction

This method should be performed simultaneously with bone and joint reconstruction, including the reconstruction of patellar ligament and attachment, joint capsule repair and other tissues related to joint stability, and it is recommended to complete soft tissue coverage in one phase as far as possible.

### *Adjuvant Chemotherapy*

Adjuvant chemotherapy after limb‐salvage surgery is an important component of the treatment of osteosarcoma. Surgery combined with neoadjuvant and adjuvant chemotherapy can improve the clinical efficacy of patients with conventional osteosarcoma^57^.

The aim of adjuvant chemotherapy is to eliminate subclinical lesions, reduce or delay distant metastasis and recurrence, and improve the efficacy of limb‐salvage therapy. The rate of tumor necrosis should be determined according to the pathological samples after the operation, and the efficacy of neoadjuvant chemotherapy should be evaluated to determine the adjuvant chemotherapy. To be sure the tumor necrosis rate and imaging results of the assessment is not exactly the same. The tumor necrosis rate for III‐IV level (necrosis area of ≥90%) patients with postoperative adjuvant chemotherapy can continue to use the neoadjuvant chemotherapy regimens, while the necrosis rate of I‐II level (necrosis area of < 90%), postoperative adjuvant chemotherapy need to adjust the chemotherapy regimen, but no decisions need to be made about whether to help improve overall survival. Postoperative adjuvant chemotherapy is generally no less than three cycles.

## Children Limb‐Salvage Surgery

### *Limb‐Salvage Surgery with Epiphyseal Preservation*

The 10‐year limb‐salvage rate of epiphyseal preservation was 90%–97%^58^, and the knee function of musculoskeletal tumor society (MSTS) after bone epiphysis preserving surgery was more than 90%. The latest clinical study reported that the local recurrence rate was about 7%^35^, ^36^, ^59^, ^60^.

This technique is suitable for children with osteosarcoma of the diaphysis or epiphysis, and neoadjuvant chemotherapy is effective. Preoperative evaluation of the relationship between tumor edge and epiphysis plate and epiphysis should be performed based on MRI. At present, the San Julian imaging method is widely used to determine the invasion of epiphyseal bone tumors in children. Type I is the absolute indication when the tumor is adjacent to the epiphyseal plate and the distance between the tumor edge and epiphyseal plate is more than 2 cm. Type II: when the distance between tumor and epiphyseal plate is less than 2 cm or adjacent. Type III is a part of the contact between the tumor and epiphyseal plate, which is more than 2 cm away from subchondral bone of the joint end. Types II and III are relative indications^61^, ^62^. In addition, it is not recommended to damage the epiphysis corresponding to the healthy side in order to balance the limb length.

### *Extendable Tumor Endoprosthesis*

This method is applicable to the bone defect after the resection of an osteosarcoma of the distal femur or the proximal tibia in children in the developmental stage, and the residual growth capacity is expected to be <4cm.The expected growth capacity of the limbs is calculated by referring to the methods of Anderson and Paley^37^, ^49^, ^63^.

Long‐term retrospective studies showed that this method had a higher incidence of complications, with the most common one being soft tissue complication (46%), followed by structural failure of the prosthesis (28%), infection (17%), and aseptic loosening (8%). The average extension was 4.4 times, and the average treatment of related complications was 2.5 times^62^.

### *Semi‐Joint Prosthesis Replacement*

This method is suitable for reconstruction of defects after resection of osteosarcoma of the distal femur and proximal tibia in children younger than 11 years old. A semi‐knee prosthesis with biaxial motion trajectory can theoretically reduce the wear of the metal prosthesis on tibial articular cartilage in children^62^.

## Postoperative Management and Prevention of Complications

Complications of any type of limb‐salvage reconstruction are common, with an overall incidence of 20%–30%^47^. Chronic disease status, systemic chemotherapy, nutrient deficiency, and coagulation system disorders can increase the incidence of complications. At the same time, mechanical or biological factors, such as reconstruction of prosthesis or allograft bone, will also bring a high incidence of local complications in limb‐salvage treatment. Severe periprosthetic infection and local tumor recurrence will lead to the failure of limb‐salvage treatment.

### *Infection*

The risk of local infection after limb‐salvage surgery exists for a long time, the postoperative infection rate is 8%–15%, with the most common being staphylococcal infection^47^.

1. Allograft bone: with an infection rate from 9% to 25%. Recently reported long‐term clinical studies showed an effective rate of 18% after debridement and antibiotic treatment. In 72% of the cases, allograft bone was removed and artificial body weight was used, and the reinfection rate was 12%^64^.
2. Artificial joint prosthesis: the infection rate of lower limb tumor endoprostheses is 8%–10%, with most of the infections occurring within 2 years post operation, 70% of the deep infections occurring within 12 months post operation. Once infection occurred, the amputation rate was 23.5%–87%^65^.

In the case of neoadjuvant chemotherapy, extensive resection and long‐segment tumor metal prosthesis implantation are the high risk factors for postoperative limb‐salvage infection. The use of antibiotics is recommended according to the class of wound. The drug is selected according to the guiding principles for the clinical application of antibiotics (2015 edition). It is suggested to refer to the wound drainage time when calculating the usual time of antibiotics.

### *Non‐Union of Allograft Bone and Fracture*

The incidence of non‐union and fracture of allograft bone is 12%–63% ^66^ and 17%–34%^62^, respectively. Risk factors include age over 18 years old, length of allograft bone over 15cm, radiation sterilization, simple intramedullary nail fixation or locking intramedullary nail fixation, and diaphysis transplantation. Combined autologous vascularize fibula graft is an effective way to reduce and prevent bone non‐union and fracture^48^, ^56^.

### *Prosthesis Looseness and Mechanical Failure of the Prosthesis*

Aseptic loosening of the prosthesis intramedullary needle is the main complication of distal femur tumor endoprosthesis replacement, and the incidence rate is 5%–11%^29^, ^46^. The application of new rotatable axis prosthesis, sagittal radian of femoral bone marrow needle, biological fixation of intramedullary needle, biological coating and other technologies have significantly reduced the loosening rate of the intramedullary needle of tumor endoprostheses compared with the simple hinge type.

The mechanical failure rate of the prosthesis is low, about 3%‐6%. The component fracture of the prosthesis, dislocated hinge device, and damage of the gasket are all defined as mechanical failure of the prosthesis^29^, ^45^, ^46^, ^67^, ^68^.

### *Local Recurrence of the Tumor*

Limb‐salvage treatment has the risk of local tumor recurrence, and the local recurrence rate is about 5.4%~10%. Local recurrence of osteosarcoma after limb salvage has an impact on the overall survival rate of the patients. The 5‐year tumor‐free survival rate is 10%–40%. The prognosis of patients with recurrence within 2 years after operation of conventional high‐grade osteosarcoma is poor^20^, ^21^, ^34^.

Multi‐factor analysis showed that the risk factors for local recurrence of osteosarcoma were failure to achieve safe surgical margin, poor histological response to chemotherapy and tumor growth during chemotherapy^32^, ^33^. Both amputation and limb‐salvage operation can be used again as treatment options for local recurrence of limb‐salvage surgery^19^, ^69^, ^70^. It is recommended that the resection range of recurrent lesions be at least l cm^33^ beyond the normal tumor margins. Recurrent lesions >5 cm with metastases were independent factors for poor prognosis^31^.

## Efficacy Evaluation of Limb‐Salvage Therapy

### *Limb Function*

The Musculoskeletal Tumor Society (MSTS) efficacy scoring system for limb‐salvage surgery is recommended^71^, ^72^. This scoring system is easy to use and can reflect the functional level of the affected limb and the whole patient. The results are repeatable and reliable.

### *Tumor Control*

Including local and systemic control, it is recommended to use the efficacy evaluation standard of solid tumor version 1.1, and there is no high‐level evidence at present.

## Rehabilitation Guidance

### *Functional Exercise*

Take the active exercise as the main activity, the passive exercise as the auxiliary. In addition to local fixation for tendon reconstruction, functional exercise was feasible 24 h after surgery. The specific method of functional exercise should be determined according to the surgical site and reconstruction method^73^, ^74^.

### *Relationship with Postoperative Chemotherapy*

Adjuvant chemotherapy after limb‐salvage surgery is an important part of the treatment of conventional osteosarcoma. Adjuvant chemotherapy can be administered after wound healing, and chemotherapy is recommended to be administered within 3 weeks after surgery. Studies have shown that delay in postoperative chemotherapy, especially in patients with poor histologic response to non‐adjuvant chemotherapy, increases the risk of local recurrence^25^, ^75^, ^76^, ^77^, ^78^, ^79^. Chemotherapy should not be given to patients with acute postoperative infection or wound non‐union. There is no clear guidance on whether adjuvant chemotherapy can be used in chronic infection, but individualized treatment is feasible.

## Follow‐Up Recommendations

The patients were followed up every 3 months for the first 2 years after limb‐salvage treatment. In the third year, the patients were followed up every 4 months. In the fourth and fifth years, the follow up was every 6 months. Eventually, the patients were followed up once a year from the fifth year of the 10^th^ year follow‐up period^2^.

## List of Names of Consultant Specialists

Jing‐ping Bai, Wen‐zhiBi, Lin Cai, Zheng‐dong Cai, Tong‐wei Chu, Yang Dong, Wei Guo, Zheng Guo, Yong‐qiang Hao, Hong‐bo He, Yong‐cheng Hu, Ling Jiang, Zhi‐hong Li, Hao‐miao Li, Jia‐zhen Li, Jian‐min Li, Ya‐ping Li, Jian‐hua Lin, Xiao‐hui Niu, Guo‐fan Qu, Guan‐ning Shang, Zeng‐wu Shao, Jing‐nan Shen, Xiao‐dong Tang, Chong‐qi Tu, Guo‐wen Wang, Jin Wang, Zhen Wang, Su‐jia Wu, Jian‐ru Xiao, Zhi‐ping Yang, Zhao‐ming Ye, Zong‐sheng Yin, Xiu‐chun Yu, Chun‐lin Zhang, Guo‐chuan Zhang, Qing Zhang, Wei‐bin Zhang, Yong Zhou.
